# Structural, electrical, and optical properties of Ti-doped ZnO films fabricated by atomic layer deposition

**DOI:** 10.1186/1556-276X-8-108

**Published:** 2013-02-27

**Authors:** Zhi-Yuan Ye, Hong-Liang Lu, Yang Geng, Yu-Zhu Gu, Zhang-Yi Xie, Yuan Zhang, Qing-Qing Sun, Shi-Jin Ding, David Wei Zhang

**Affiliations:** 1 State Key Laboratory of ASIC and System, Department of Microelectronics, Fudan University, Shanghai, 200433, China

**Keywords:** TZO films, ALD growth rates, Structure characteristics, Optical properties, Electrical properties

## Abstract

High-quality Ti-doped ZnO films were grown on Si, thermally grown SiO_2_, and quartz substrates by atomic layer deposition (ALD) at 200°C with various Ti doping concentrations. Titanium isopropoxide, diethyl zinc, and deionized water were sources for Ti, Zn, and O, respectively. The Ti doping was then achieved by growing ZnO and TiO_2_ alternately. A hampered growth mode of ZnO on TiO_2_ layer was confirmed by comparing the thicknesses measured by spectroscopic ellipsometry with the expected. It was also found that the locations of the (100) diffraction peaks shift towards lower diffraction angles as Ti concentration increased. For all samples, optical transmittance over 80% was obtained in the visible region. The sample with ALD cycle ratio of ZnO/TiO_2_ being 20 had the lowest resistivity of 8.874 × 10^−4^ Ω cm. In addition, carrier concentration of the prepared films underwent an evident increase and then decreased with the increase of Ti doping concentration.

## Background

The application of transparent conductive oxide (TCO) has been found in many areas such as liquid crystal displays, touch panels, and organic light-emitting diodes because of its excellent conductivity and high transmittance for visible light [1-4]. At the moment, indium tin oxide film is the most popular material and has been widely used in many optoelectronic devices. Although it has excellent transparency (greater than 85% transparency in visible spectrum) together with high conductivity (resistivity around 2 to 4 × 10^−4^ Ω cm) [5], research on alternative materials is urgent due to the relative scarcity and high cost of indium.

Several alternative TCO materials have been investigated extensively recently. Among them, ZnO seems to be one of the ideal choices due to its low cost. As is well known, ZnO is a II-VI group semiconductor material containing high concentration of native defects, which typically include oxygen vacancies or zinc interstitials. Thus, pure ZnO has excellent conductivity. However, pure ZnO thin films are not very electrically and chemically stable at high temperature [6]. Fortunately, the performance of ZnO thin films can be improved by appropriate impurity doping [7]. For example, it has been reported that Al-doped ZnO film fabricated by atomic layer deposition (ALD) has as high as 80% to 92% transmittance in the visible range and low resistivity around 4 × 10^−3^ Ω cm [8]. What is more, as is reported by Lin et al., Zr-doped ZnO thin films grown by atomic layer deposition with sapphire substrates have wonderful transparency (>92%) for visible light and high carrier concentration (2.2 × 10^20^) [9].

Among the variety of metallic element-doped ZnO films, Ti-doped ZnO films have been investigated recently for their unique electrical, magnetic, and sensing properties. In some previous studies, a number of fabrication techniques such as sputtering, pulsed laser deposition, and chemical vapor deposition (CVD) as well as the structural, morphological, and electrical characteristics of the corresponding films [10-16] have been discussed. However, rare reports focused on Ti-doped ZnO films fabricated by ALD. Furthermore, compared with those of main group metal-doped ZnO films, the conduction mechanisms of ZnO films doped with transition metals such as Ti are still not clearly understood. So it is of greater importance to do research on Ti-doped ZnO (TZO) films grown by ALD. In this work, the effect of Ti doping concentration on the structural, optical, and electrical properties of the deposited TZO films was systematically studied by spectroscopic ellipsometry, X-ray diffraction, atomic force microscopy, transmission spectrometry, and Hall measurement.

## Methods

TZO thin films were deposited at 200°C in a BENEQ TFS-200 ALD reactor (Vantaa, Finland) using titanium tetraisopropoxide liquid (TTIP), diethyl zinc (DEZ), and deionized (DI) water. TTIP, DEZ, and DI water were used as Ti, Zn, and O sources, respectively. The precursors TTIP and DEZ were separately held in stainless bubblers at 58°C and 18°C, respectively. High-purity quartz, thermally grown SiO_2_, and silicon served as the substrates. Before loading into the ALD reactor, the quartz glasses were ultrasonically cleaned with acetone and alcohol in sequence for 5 min, and then rinsed with DI water and dried in nitrogen. The silicon substrates were cleaned chemically using a standard Radio Corporation of America solution (New York, NY, USA) and then dipping into the diluted 5% HF solution for 1 min to remove the native oxide layer, followed by rinsing with DI water and drying in N_2_. The precursors were alternately introduced to the reactor chamber using high-purity N_2_ (>99.99%) as the carrier gas. A typical ALD growth cycle for ZnO is 0.5-s DEZ pulse/2-s N_2_ purge/0.5-s H_2_O pulse/2-s N_2_ purge, whereas for TiO_2_, it is 1.0-s TTIP pulse/5-s N_2_ purge/0.5-s H_2_O pulse/5-s N_2_ purge. The TZO films were then achieved in an ALD supercycle mode, which was defined as *N* ZnO cycles followed by one TiO_2_ cycle. Supercycles were repeated until the target number of 500 ZnO cycles was reached.

The thicknesses of TZO films were measured by spectroscopic ellipsometry (GES5E, SOPRA, Courbevoie, France) wherein the incident angle was fixed at 75° and the wavelength region from 230 to 900 nm was scanned with 5-nm steps. The crystal structures of films were obtained using an X-ray diffractometer (D8 ADVANCE, Bruker AXS, Madison, WI, USA) using Cu Kα radiation (40 kV, 40 mA, *λ* = 1.54056 Å). Atomic force microscopy (AFM) using a Veeco Dimension 3100 scanning probe microscope (Plainview, NY, USA) operated in a tapping mode provided surface morphology of the TZO thin films. To obtain the optical transmission spectra, a UV spectrophotometer (UV-3100) in a wavelength range of 200 to 900 nm at room temperature was used in the air. In addition, the electrical properties of TZO films deposited on thermally grown SiO_2_ are characterized by Hall effect measurements using the van der Pauw method.

## Results and discussion

The growth per cycle (GPC) of pure ZnO and TiO_2_ films are tested to be 0.2 and 0.025 nm/cycle, respectively. Measured thicknesses of TZO films are then listed in Table 1 together with the expected thicknesses, which are given by

(1) $$\mathrm{Thickness}=500\times \mathrm{GP}C_{\mathrm{ZnO}}+\frac{500}{N}\times \mathrm{GP}C_{\mathrm{TiO}2},$$

**Table 1:** **Summary of estimated and measured thicknesses of TZO films with *R***^**2 **^**accuracy greater than 0.995**

**Sample**	**Number**	**Number of supercycle**	**Estimated thickness (nm)**	**True thickness (nm)**
ZnO	N/A	500	100.0	106 ± 2.1
Zn/Ti = 20:1	20	25	100.8	101 ± 1.7
Zn/Ti = 10:1	10	50	101.5	95 ± 0.9
Zn/Ti = 5:1	5	100	103.0	94 ± 1.5
Zn/Ti = 2:1	2	250	107.5	84 ± 1.4
Zn/Ti = 1:1	1	500	115.0	80 ± 0.6

**Figure 1:**
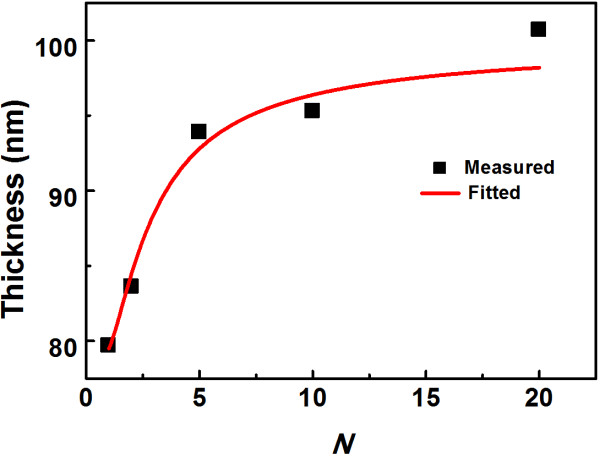
Fitting curve according to the function model is shown with a red solid line.

In Equation 1, it is assumed that the GPC for a given material has no business with the material deposited in the previous cycle. Since the GPC of ZnO is much greater than that of TiO_2_, the estimation of the film thickness is accurate provided that ZnO encounters no barrier to grow on TiO_2_. As an example, for the TZO film with *N* = 20, the measured thickness is 101 nm, which is very close to the expected one. However, with further increase of Ti doping concentration, the measured film thicknesses are found to be off-target. Especially, in the case of the sample with *N* = 1, the measured thickness was found to be around 80 nm, which was much smaller than the ideal one (115.0 nm). Thus, it is inappropriate for us to assume the GPC of ZnO on TiO_2_ to be 0.2 nm/cycle. The black squares in Figure 1 show the true thickness as a function of *N*.

To model the true growth process of ALD-ZnO film on TiO_2_ layer, a method similar to that reported by Banerjee et al. [8] was employed. The decrease of the GPC of ZnO may result from the reduced adsorption of DEZ on TiO_2_. Thus, it is appropriate to assume that the GPC of ZnO follows an exponential behavior given by

(2) $$\mathrm{GP}{C^{\prime }}_{\mathrm{ZnO}}=A\left(1-e^{-\frac{i}{n}}\right),$$

where *GPC*^′^_*ZnO*_ represents the GPC of ZnO in TZO film, *A* is the GPC of pure ZnO film, the independent variable *i* is the *i*th cycle number after TiO_2_ deposition, and the parameter *n* refers to the number of cycles it needs for *GPC*^′^_*ZnO*_ to reach 63.2% of the ideal growth rate of ZnO. According to Equation 2, the *GPC*^′^_*ZnO*_ would be close to that observed in pure ZnO films after enough number of ZnO cycles. It is also appropriate to assume that *GPC*^′^_*TiO*2_ remains unchanged throughout the whole process since TiO_2_ is always deposited on ZnO. Considering all the assumptions above, the total thickness of the film can be given by

(3) $$T=\left[\sum_{i=1}^{N}{\mathrm{GPC}}_{\mathrm{ZnO}}^{0}\left(1-e^{-\frac{i}{n}}\right)+t\right]\times \frac{500}{N},$$

where *T* denotes the total thickness and the constant *t* is the GPC of TiO_2_. Using this function model to fit the measured data, the parameter *n* can be calculated to be approximately 1 while *t* is approximately 0.024 nm/cycle. Thus, it can be concluded that TiO_2_ encounters little barrier to grow on ZnO.

**Figure 2:**
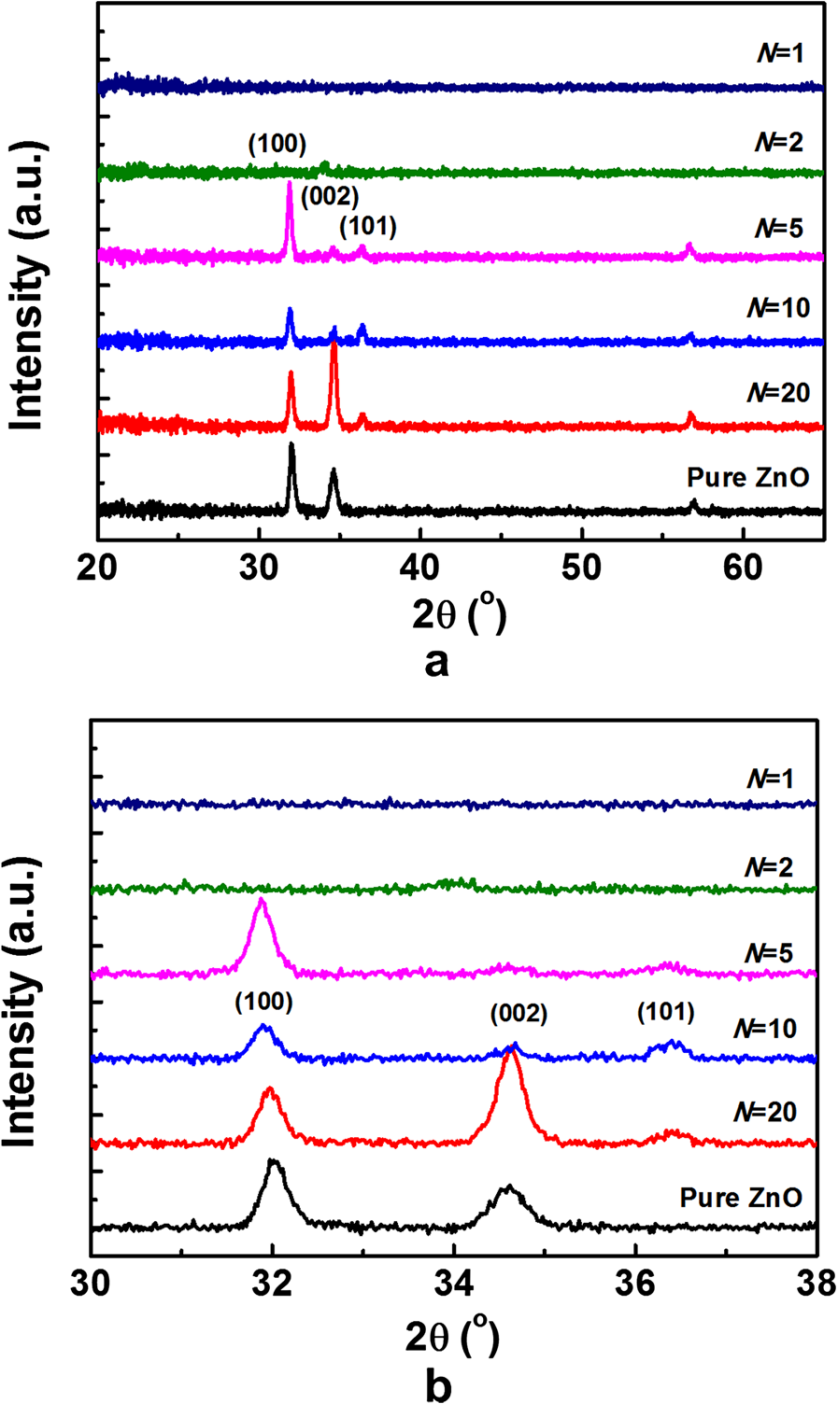
**XRD patterns for TZO films deposited on quartz for 2*θ*.** (**a**) 20° to 65° and (**b**) 30° to 40°.

Figure 2 shows the XRD patterns of as-deposited TZO films on quartz. As is displayed in Figure 2a, the crystallinity of the films depends on the *N*. No phases related to TiO_2_ or Zn_2_TiO_4_ are detected in the scanning range. Usually, the [002] direction, i.e., the *c*-axis, is the preferential orientation commonly occurring in pure ZnO films and doped ZnO films prepared by other fabrication techniques such as sol–gel, CVD, and sputtering [10]. However, in the current samples, the (100) peak gradually becomes dominant and the (002) peak turns to be weaker as Ti doping concentration increases. The (100) peak reaches a maximum for the sample with *N* = 5. However, no peak can be observed in the samples with *N* = 2 and 1, indicating that the TZO films become amorphous with too much Ti doping. It is well known that the (002) plane of ZnO consists of alternate planes of Zn^2+^ and O^2−^ and thus is charged positively or negatively, depending on surface termination. On the other hand, the (100) plane is a charge neutral surface consisting of alternate rows of Zn^2+^ and O^2−^ ions on the surface. Thus, it is conceivable that the layer-by-layer growth during ALD may cause the Ti^4+^ ions to disturb the charge neutrality of the (100) plane, thereby affecting its surface energy and causing its preferential growth [8].

In addition, the locations of the (100) diffraction peaks shift towards lower diffraction angles as Ti concentration increases, as shown in Figure 2b. To understand this phenomenon, it is worthwhile to notice that the valence of Ti tends to be +4 in the TZO films made by atomic layer deposition. Along the [100] direction, the film layer is composed of the line of Zn^2+^ ions or the line of O^2−^. If Ti^4+^ ions take the place of Zn^2+^ sites, the repulsive force in this direction would increase because of extra positive charge. This effect can enlarge the interplanar spacing along the [100] direction, thus leading to the observed decrease of the diffraction angle.

**Figure 3:**
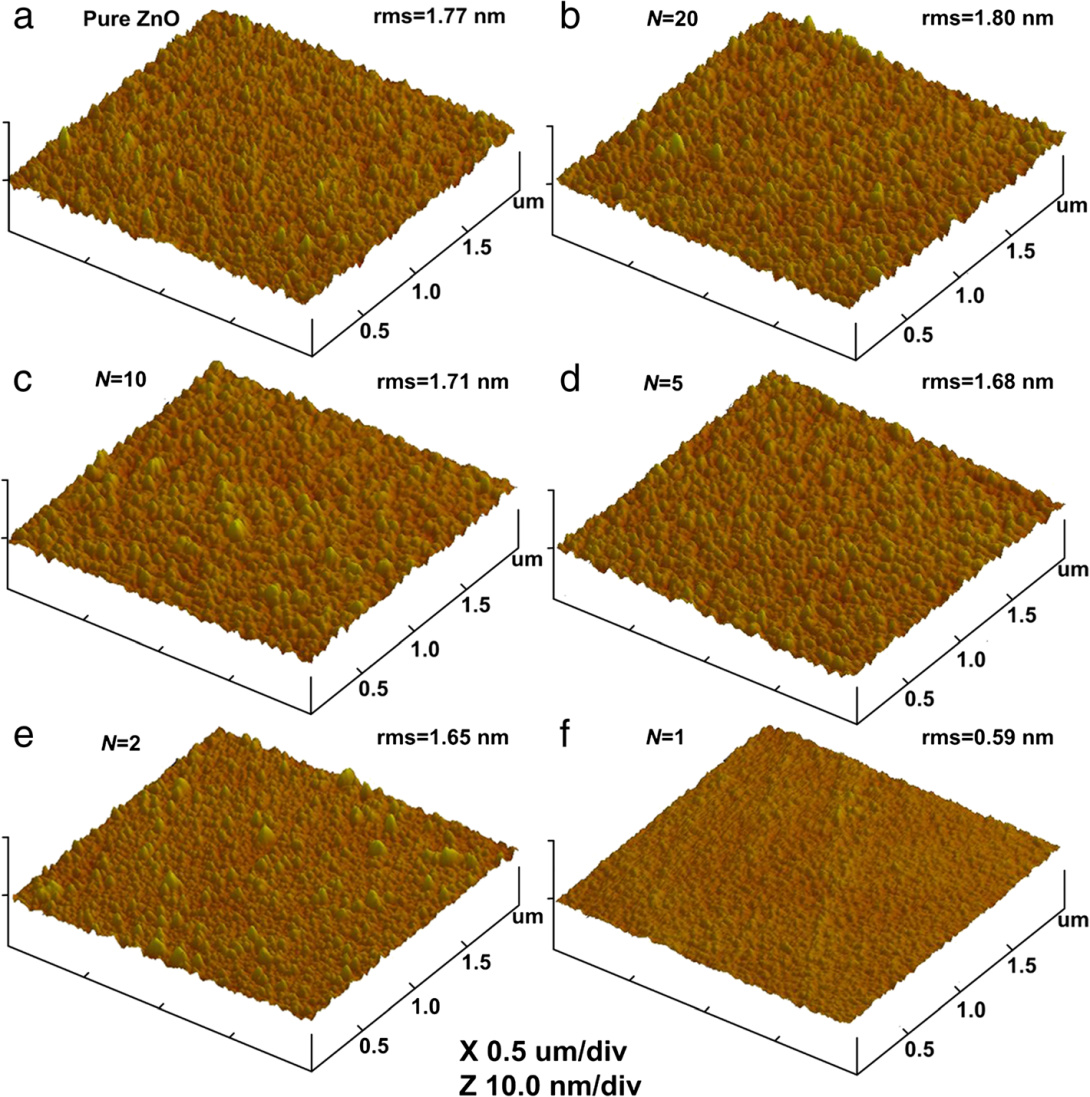
**AFM images of TZO films with rms surface roughness in a scan area of 2 × 2 μm**^**2**^**.**

The AFM images of the films deposited on silicon substrate were measured to further characterize the effect of Ti doping concentration on the surface morphology of TZO films. Figure 3 shows the AFM images of these films and their root mean square (rms) surface roughness in a scan size of 2 × 2 μm^2^. It was found that the rms roughness value of the films except for the sample with *N* = 1 is in the range of 1.65 to 1.80 nm. The surfaces of these films are evidently smoother than those deposited by RF reactive magnetron sputtering [10]. It highlights the potential use of TZO films made by ALD as TCO, where precise control over film uniformity and smoothness is required. The film with *N* = 1 shows the lowest surface roughness with its rms roughness value to be 0.59 nm. In addition, no etching effect on the film is found in the experiment [17].

**Figure 4:**
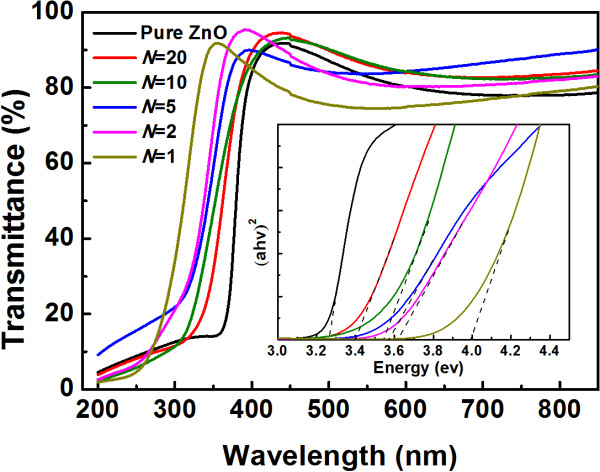
**Transmission spectra of TZO films with various Ti concentrations.** The inset shows the plot of (*αhv*)^2^ versus *hv*.

Figure 4 displays the transmission spectra of TZO films grown on quartz. It is obvious that an average optical transmittance more than 80% in the visible range is obtained for the samples with *N* from 20 to 2, which is valuable for TCO applications such as liquid crystal displays. The relatively low transmission for the sample grown with *N* = 1 resulted from the high concentration of Ti in the TZO films. Moreover, all the films show a sharp absorption edge in the ultraviolet range, which shifts to the lower wavelength side with Ti concentration increase. The optical band gap of TZO thin films can be calculated according to the transmission spectra. As a direct-band gap material [18], it is reasonable to assume that the absorption coefficient (*α*) is proportional to − ln(*T*), where *T* is the optical transmission. According to the Tauc relationship, the relation between the optical band gap (*E*_g_) and absorption coefficient is given by [19]

(4) $$\mathrm{\alpha hv}∼{\left(\mathrm{hv}-E_{g}\right)}^{1/2},$$

where *h* is Planck's constant and *v* is the frequency of the incident photon. The *E*_g_ of the TZO films can be obtained by drawing the plot of (*α* × *hv*)^2^ versus the photon energy (*hv*) and extrapolating a straight line portion of this plot to the axis of photon energy, as is indicated in the inset of Figure 4. It can be found that the band gap energy increases from 3.26 eV for pure ZnO film to 3.99 eV for the film with *N* = 1. The widening of band gaps with the increase of titanium concentration is generally attributed to the Burstein-Moss band-filling effect. Excessive carriers induced by the doped Ti would fill the conduction band edge, so the optical band gap is widened [20,21].

**Figure 5:**
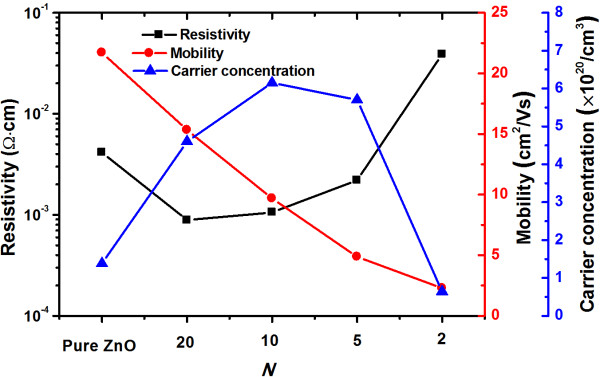
**Resistivity, mobility, and carrier concentration of the TZO films deposited on thermally grown SiO**_**2**_**.**

To investigate the electrical properties of the TZO thin films, Hall measurements are carried out at room temperature. The thermally grown SiO_2_ was chosen as the substrate since the substrate needs to be insulative. The dependence of carrier density, resistivity, and mobility on Ti contents in the TZO films is shown in Figure 5. It should be noted that the resistivity of the sample with *N* = 1 is so large that its mobility and carrier concentration cannot be measured accurately. As is displayed, the resistivity, mobility, and carrier concentration for pure ZnO films prepared by ALD are 2.14 × 10^−3^ Ω cm, 1.4 × 10^20^ cm^−3^, and 22.5 cm^2^/V · s, respectively. The resistivity of the TZO film with *N* = 20 at first drops to a minimum value of 8.874 × 10^−4^ Ω cm and then goes up with the increase of the Ti contents. It suggests that the conductivity of ZnO film can be improved significantly with appropriate Ti doping concentration. On the other hand, the maximum carrier concentration of 6.2 × 10^20^ cm^−3^ is achieved for the sample with *N* = 10, which is higher than that reported by Park and Kim [22]. However, carrier concentration of the TZO film undergoes an abrupt drop when more Ti impurities are introduced into the TZO film. The decrease in the carrier concentration can be interpreted as follows: As the Ti doping concentration continues to increase, some titanium atoms tend to aggregate near grain boundaries to form TiO_2_ instead of taking the place of Zn^2+^ to generate more free carriers [23]. The widening of band gap is also generally considered as a dominant mechanism contributing to the decrease of carrier concentration [20,21]. In addition, the mobility of TZO films decreases from 21.7 cm^2^/s for pure ZnO to 2.3 cm^2^/s for the sample with *N* = 2. The decrease in mobility is apparently due to the increase of carrier scattering, the deterioration in the crystalline quality, and formation of TiO_2_ at the grain boundaries.

## Conclusions

Ti-doped ZnO thin films with the thickness of around 100 nm were prepared by ALD at 200°C. The fact that film thicknesses measured by spectroscopic ellipsometry were thinner than expected for samples with ALD cycle ratio of ZnO/TiO_2_ less than 10 suggested a hampered growth mode of ZnO on TiO_2_ layer. TZO films synthetized by ALD crystallized preferentially along the [100] direction. High transparency (>80%) in the visible region was obtained, and the band gap of the TZO films increased with increasing Ti doping concentration due to the Burstein-Moss effect. It was observed that the resistivity of TZO film had a minimum value of 8.874 × 10^−4^ Ω cm when the ALD cycle ratio between ZnO and TiO_2_ was 20.

## Competing interests

The authors declare that they have no competing interests.

## Authors’ contributions

The experiment was designed by ZYY and HLL and revised by QQS, SJD, and DWZ. The fabrication of TZO films was carried by ZYY and YG. The characteristics of the films were tested and analyzed by ZYY with the help from YG, YZG, ZYX, and YZ. ZYY prepared the manuscript, and HLL gave a lot of help with the draft editing. All of the authors have read and approved the final manuscript.
